# Percutaneous pulmonary valve implantation: Pre- and post-procedural RVOT and coronary artery relationship assessed by CT

**DOI:** 10.1016/j.ijcha.2023.101224

**Published:** 2023-05-20

**Authors:** Marguerite E. Faure, Ricardo P.J. Budde, Annemien E. van den Bosch, Jeroen M. Wilschut, Tim ten Cate, Anthonie L. Duijnhouwer, Jolien W. Roos-Hesselink, Alexander Hirsch

**Affiliations:** a Dep. of Radiology and Nuclear Medicine, Erasmus Medical Center, University Medical Center Rotterdam, Rotterdam, the Netherlands; b Dep. of Radiology, AZ Monica, Antwerp, Belgium; c Dep. of Cardiology, Erasmus Medical Center, University Medical Center Rotterdam, Rotterdam, the Netherlands; d European Reference Network for Rare and Low Prevalence Complex Diseases of the Heart, the Netherlands; e Dep. of Cardiology, Radboud University Medical Center, Nijmegen, the Netherlands

**Keywords:** Percutaneous pulmonary valve intervention, Computed tomography, Coronary arteries, Right ventricular outflow tract

Patients with congenital heart diseases involving the right ventricular outflow tract (RVOT) often develop RVOT dysfunction despite undergoing surgical correction early in life. Percutaneous pulmonary valve implantation (PPVI) is an effective treatment [1], [2], [3] that encompasses inserting a balloon-expandable stent mounted valve, frequently preceded by pre-stenting to provide an adequate landing zone, to prevent valvular fracture and paravalvular leak. A serious complication of PPVI is coronary artery compression caused by stent expansion, which can result in myocardial ischemia or infarction and may require emergency surgery [4]. This risk is classically evaluated before implantation by invasive aorto-coronary angiography with simultaneous balloon inflation in the pulmonary landing zone. Recent studies suggest cardiac CT angiography (CTA) can also identify which patients are at risk for coronary artery compression [4], [5]. Cardiovascular magnetic resonance (CMR) on the other hand, may assist in patient selection and counseling families prior to PPVI, but has a more limited role in excluding coronary artery compression [6]. To our knowledge, no imaging studies are available concerning post-procedural conduit expansion and/or deviation and how this might affect coronary anatomy and compression. Transthoracic echocardiography (TTE) is the first-line modality used for post-procedure surveillance of PPVI patients [7]. CT (and CMR) are mostly used for further characterization of valvular abnormalities identified at TTE and are not routinely performed after PPVI. This probably explains why limited data is available about post-procedural findings regarding the RVOT and its relationship with the coronary arteries.

In this multicenter exploratory cross-sectional observational study, the relationship between the coronary arteries and RVOT before and after PPVI was assessed using CTA to evaluate possible relevant changes in RVOT to coronary distance and coronary lumen diameter to study the reliability of pre-procedural CT risk assessment.

Patients aged ≥ 18 years, that had undergone PPVI and had a pre-procedural CTA were prospectively invited for a follow-up study, including CTA. Exclusion criteria were those applicable for CT with administration of intravenous contrast material including patients that would need pre-hydration prior to contrast administration, unwillingness to be informed about unrequested findings on CT and mentally incapacitated adults. Image acquisition was performed on a dual source CT scanner (SOMATOM Force or Drive, Siemens, Erlangen). A prospectively ECG-triggered wide-pulsing-window sequential CTA was performed (fixed tube voltage of 120 kV, reference effective tube load 180 mAs). A single contrast administration protocol was used in which 100 ml of contrast was given with an Iodine concentration of 320 mg/ml (Visipaque 320®). The relationship between RVOT and the nearest coronary artery was assessed. In all patients this was the left main or left anterior descending artery. In the pre- and post PPVI CTAs, the minimum distance to the valve was measured at the same level and in the same way using double oblique reconstructed images as described before [5] (Fig. 1A). Furthermore, coronary morphology and lumen diameter stenosis of the coronary artery closest to the RVOT were visually assessed. Wilcoxon signed-rank test was used to compare measurements before and after PPVI, since data were not normally distributed.

**Fig. 1A:**
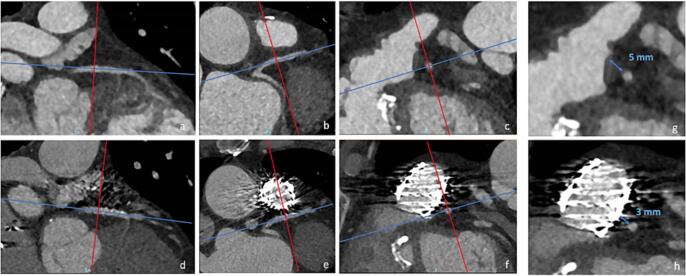
CTA before (a-c) and after PPVI (d-f). All measurements were performed using double oblique reconstructed images. Coronary artery morphology, patency and the relationship with the RVOT was assessed (g,h).

In total, 89 patients underwent PPVI between 2007 and 2019 in the Erasmus Medical Center of which 62 patients had a CTA prior to PPVI. During follow-up two patients died and two underwent valve explantation. Further, due to the recent Coronavirus pandemic at the time of the study and the rather precarious state of health of these patients, a relatively large number of patients were unwilling to come to the hospital. Finally, 27 of 58 patients met the inclusion criteria and gave informed consent and underwent post-procedural CTA, after ethics committee approval (COVER, MEC-2020-0257). Of the 27 paired scans, three post PPVI CTAs showed too much metal artefacts for proper evaluation, so 24 patients were included for analysis. Pre-procedural clinical and echocardiographic analysis are shown in Table 1. Mean age of the patients was 35 ± 13 years and 71% (17/24) was male. The majority of the underlying pathology was Tetralogy of Fallot, 46% (11/24) and aortic valve disease for which a Ross procedure was performed, 21% (5/24). In 75% (18/24) of patients, the RVOT landing zone consisted of a pulmonary homograft conduit. Only 2 patients had a stented bioprosthetic valve that generally does not expand. Main reason for PPVI was in 67% a progressive pulmonary stenosis, in 25% pulmonary insufficiency, and in 8% a combination. Median time between both CTAs was 5.5 (3.5–6.9) (25-75th percentile) years (time between pre-procedural CTA and PPVI was 0.3 (0.3–0.5) years and time between PPVI and post-procedural CTA was 5.2 (3.6–8.3) years). In 20 patients a Melody valve (Medtronic, Minneapolis, Minnesota, USA) was implanted and in 4 patients a SAPIEN transcatheter heart valve (Edwards Lifesciences, Irvine, California) (Table 1). Median coronary to RVOT distance before PPVI was 4.5 mm (2.0–9.0) and after PPV 4.0 (2.3–8.8) (p-value 0.27) (Fig. 1B). When the patients were divided into different categories based on the distance between coronary artery and RVOT, no differences were found between pre- and post-procedural CTAs: before PPVI the distance was < 5 mm in 50% (12/24) of the patients and post PPVI in 54% (13/24) (p = 0.70, Table 1). None of the coronary arteries showed significant luminal narrowing after PPVI.

**Table 1:** Baseline characteristics of the patients that underwent percutaneous pulmonary valve implantation and patients divided into different categories based on the minimum distance between right ventricular outflow tract and nearest coronary artery.

	**N = 24**
Age (years)	35 ± 13
Male	17 (71%)
Body mass index (kg/m^2^)	24 ± 4
New York Heart Association class I/II	21 (88%)
Underlying pathology	
Tetralogy of Fallot	11 (46%)
Ross procedure for aortic valve disease	5 (21%)
Transposition of the great arteries	3 (13%)
Pulmonary atresia with VSD	3 (13%)
Congenital pulmonary stenosis	2 (8%)
Medications pre PPVI	
β-blocker	7 (29%)
ACE inhibitor/angiotensin receptor blocker	2 (8%)
Diuretics/MRA	3 (13%)
Direct oral anticoagulants or coumarin derivates	3 (13%)
Aspirin	2 (8%)
Echocardiography pre PPVI	
Moderate or greater impairment of LV systolic function	2 (8%)
TAPSE (mm)	16 ± 4
Right ventricular fractional area change (%)	36 ± 14
Right atrial area (cm^2^)	25 ± 12
Moderate or greater aortic regurgitation	1 (4%)
Moderate or greater mitral valve regurgitation	0
Moderate or greater tricuspid regurgitation	9 (38%)
Mild or greater aortic-, mitral- or tricuspid valve stenosis	0
RVOT landing zone	
Pulmonary homograft conduit	18 (75%)
Native	1 (4%)
Bioprosthetic (Melody-in-Melody)	2 (8%)
Other	3 (13%)
Indication PPVI	
Valve stenosis	16 (67%)
Valve insufficiency	6 (25%)
Combination	2 (8%)
Implanted valve type	
Melody valve	20 (83%)
SAPIEN valve	4 (17%)
Median time between CTA – PPVI (years)	0.3 (0.3–0.5)
Median time between PPVI – CTA (years)	5.2 (3.6–8.3)
Median time between both CTAs (pre and post) (years)	5.5 (3.5–6.9)
Minimum distance between RVOT and coronary artery before PPVI	
<5 mm	12 (50%)
5–9 mm	7 (29%)
10–14 mm	3 (13%)
≥15 mm	2 (8%)
Minimum distance between RVOT and coronary artery after PPVI	
<5 mm	13 (54%)
5–9 mm	7 (29%)
10–14 mm	1 (4%)
≥15 mm	3 (13%)

Minimum distance between RVOT and coronary artery.

Data is presented as number (percentage), mean ± standard deviation or median (25th – 75th percentile). ACE = Angiotensin-Converting Enzyme; CTA = CT angiography; LV = left ventricular; MRA = mineralocorticoid receptor antagonists; PPVI = Percutaneous pulmonary valve implantation; RVOT = right ventricular outflow tract; TAPSE = Tricuspid annular plane systolic excursion; VSD = ventricular septum defect.

**Fig. 1B:**
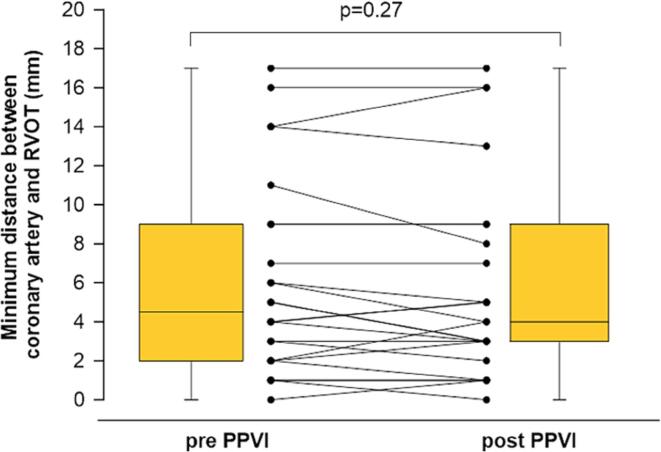
Boxplot showing the smallest distance between the nearest coronary artery and RVOT before and after PPVI.

Although coronary distance prior to PPVI can adequately be measured with CTA, conduit expansion during PPVI is not always predictable, especially when the RVOT is calcified or scarred. This study is the first that directly compares pre- and post-procedural CTA findings. Our study, although relatively small, suggests that there is no relevant change in coronary distance and coronary lumen diameter after successful PPVI.

There are some limitations to the study. First, only a limited number of patients was included. Second, since only patients that underwent PPVI were included, there can be a selection bias excluding patients that were considered too high-risk as well as those that underwent PPVI but died due to complications. Third, due to small sample size and the observational nature of this study, our findings should be validated in larger multicentric studies with longer follow-up duration.

In conclusion, conduit expansion does not seem to affect the relationship between the RVOT and coronary arteries after successful PPVI. This strengthens the reliability of pre-procedural CTA for risk assessment of coronary compression in patients undergoing PPVI.

All authors take responsibility for all aspects of the reliability and freedom from bias of the data presented and their discussed interpretation.

## Declaration of Competing Interest

The authors declare that they have no known competing financial interests or personal relationships that could have appeared to influence the work reported in this paper.
